# Antibacterial prescribing for acute upper respiratory infections in outpatients in Finland, 2017–2021: a Finnish registry study

**DOI:** 10.1093/jacamr/dlag017

**Published:** 2026-02-23

**Authors:** Lotta Mononen, Eveline Otte Im Kampe, Jukka Ollgren, Reetta Huttunen, Emmi Sarvikivi

**Affiliations:** Tampere University Hospital, Infectious Diseases Unit, The Wellbeing Services County of Pirkanmaa, Tampere, Finland; Department of Public Health, Finnish Institute for Health and Welfare, Helsinki, Finland; Department of Public Health, Finnish Institute for Health and Welfare, Helsinki, Finland; ECDC Fellowship Programme, Field Epidemiology Path (EPIET), European Centre for Disease Prevention and Control (ECDC), Solna, Sweden; Department of Public Health, Finnish Institute for Health and Welfare, Helsinki, Finland; Tampere University Hospital, Infectious Diseases Unit, The Wellbeing Services County of Pirkanmaa, Tampere, Finland; Department of Public Health, Finnish Institute for Health and Welfare, Helsinki, Finland

## Abstract

**Background:**

Antibiotic use is the main driver of antimicrobial resistance and adverse events. The aim of our study was to investigate the magnitude of prescribing antibacterials to outpatients with an acute upper respiratory infection (AURI) in Finland.

**Materials and methods:**

For the period 2017–2021, we linked data on antibacterial prescriptions (ATC group J01) from the Finnish Social Insurance Institution with disease data from Primary Care Outpatient Treatment Register and excluded diagnoses for which antibacterial treatment is indicated. We calculated the percentage of visits associated with at least one antibacterial prescription and performed univariate and multivariate logistic regression to assess the effect of year, demographic factors, AURI and selected underlying conditions.

**Results:**

Overall, 9% of all outpatient AURI diagnoses, most of which are of viral origin, were prescribed antibacterials in the public healthcare sector. This proportion decreased from 11% in 2017 to 6% in 2021 and increased with age. Women were slightly more likely to obtain a prescription than men (adjusted probability 1.09; 95%CI 1.08–1.11). Chronic pulmonary disease was associated with increased prescribing for AURIs in the multivariate analysis (adjusted probability 1.93; 95%CI 1.84–2.03), and acute tonsillitis had the highest likelihood of antibacterial prescribing compared with other AURIs (adjusted probability 14.70; 95%CI 13.41–16.12).

**Conclusions:**

Our research indicates that despite previous antimicrobial stewardship efforts, prescribing antibacterials for non-bacterial infections is still common in Finland. Nonetheless, the percentage of inappropriate prescribing decreased over time. Prescribing habits seem to be influenced by patient demographics and underlying chronic illnesses.

## Introduction

Antimicrobial usage is identified as the main cause for antimicrobial resistance (AMR) and can result in negative outcomes.^1–6^ In 2019, bacterial AMR led directly to 1.27 million deaths globally.^7^ In the EU/EEA region antibiotic resistant bacteria caused annually an estimated 600 000 infections between 2016 and 2020 and >30 000 attributable deaths between 2016 and 2019.^8^ According to the European Surveillance of Antimicrobial Consumption Network (ESAC-Net), most antimicrobials are prescribed in an outpatient setting, comprising ∼87% of the total consumption in Finland in 2015.^9^

Antimicrobial stewardship aims to encourage responsible antimicrobial use through AMR monitoring and antimicrobial consumption (AMC) surveillance, healthcare personnel education and the promotion of appropriate antimicrobial prescribing. In Finland, AMC surveillance relies on sales data recorded by the Finnish Medicine Agency in line with ESAC-Net. Additionally, Finland has formulated a national action plan on AMR.^9^

Understanding current antibacterial use and factors affecting antibacterial prescribing is crucial in implementing an effective intervention strategy. Previous research indicates that a multifaceted intervention increases the use of recommended first-line antibacterials and shortens treatment durations, however, it does not affect the overall prescription rate.^10^ In Finland, with a population of 5.6 million people, 1.4 million courses of systemic antibacterials were dispensed to 870 000 outpatients in 2021 according to the pharmaceutical sales statistics.^11,12^ Two studies published in 2022 analysed the appropriateness of antibacterial prescribing for upper respiratory infections and otitis media in children from a private healthcare provider network in Finland and observed that even though the unnecessary prescription rate decreased during the study period in the years 2014–2020 it remained high at 8.8%.^13,14^

Acute upper respiratory tract infections (AURIs) are common, typically of viral origin,^15,16,17^ and do not require antibacterial treatment. To our knowledge there is no recent evidence on antibacterial prescribing for AURIs for adults and older people, nor from the public healthcare sector. The aim of our research was to investigate the magnitude and the factors affecting prescribing antibacterials for AURIs in outpatients in the public healthcare sector in Finland during 2017–2021.

## Materials and methods

Data on antibacterial prescriptions (ATC group J01) were available from the Finnish Social Insurance Institution (Kela) for 2017–2021. For the same period, outpatient visits registered in the Primary Care Outpatient Treatment Register (Avohilmo) were extracted. To be included in this study, a visit had to have at least one diagnosis with these ICD-10 codes: J00, J02, J02.8, J02.9, J03, J03.8, J03.9, J04, J04.0, J04.1, J04.2, J05, J05.0, J05.1, J06, J06.0, J06.8 or J06.9. Visits with ICD-10 codes J01, J02.0 and J03.0 were excluded from the data as these diagnoses refer to a bacterial aetiology. The diagnoses codes were analysed in three-character ICD-10 categories.

**Figure 1.**
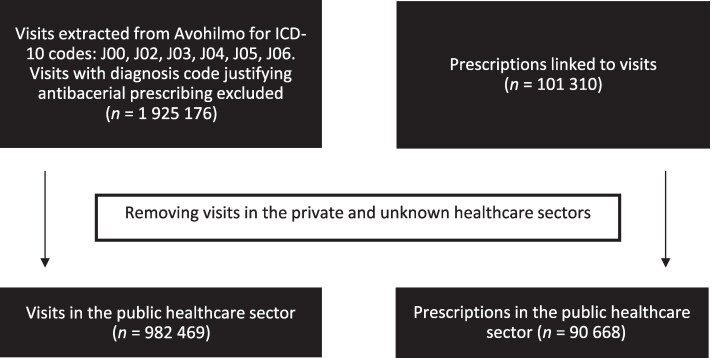
Processing flow of outpatient visit data and antibacterial prescription data in Finland, 2017–2021. Avohilmo: Primary Care Outpatient Treatment Register in Finland.

Visit data were linked to prescription data by using the patient's personal identifier, the date of visit and prescription, and the organizational identifier of the healthcare provider’s service unit. Visits with private or unknown healthcare sectors were excluded, since Avohilmo registry covers all public primary healthcare outpatient contacts in Finland during the entire study period, but the coverage of private healthcare was poor until 2020. We assumed that antibacterials were prescribed on the day of the visit. Prescriptions and linked visits with more than one diagnosis were excluded if any of the additional diagnoses would justify using an antibacterial (Figure 1). We also assessed the number of different antibacterials prescribed on a single visit.

Mean annual population data were obtained from Statistics Finland.^18^

Patients’ dates of birth were used to calculate their ages in years at the time of visit. Age groups were defined as <2, 2–4, 5–11, 12–17, 18–64 and 65+ years.

We analysed data descriptively by calculating the number of visits at the public healthcare sector, year of visit, sex, AURI type and selected underlying diagnoses. We calculated the visit rate per population by dividing the number of AURI visits by the population number. To describe visits by age group, we calculated the annual incidence of visits per 1000 population.

We calculated the percentage of visits associated with at least one antibacterial prescription by year and age group, sex, AURI type and selected underlying conditions [chronic pulmonary disease (CPD), diabetes and chronic inflammatory disease]. CPD was defined as ICD-10 codes J44–J46, J60–J70, E84.0 and C34–C39; diabetes as E10–E14 and chronic inflammatory diseases as K50–K52 and M05–M14. The underlying conditions were considered only if the corresponding diagnosis code was recorded for the AURI visit. We used univariate and multivariate logistic regression to estimate adjusted probabilities (predictive margins)^19^ with 95% confidence intervals (95%CI). The effect of underlying diagnoses and AURI type was adjusted for age group at visit, sex, year and acute nasopharyngitis diagnosis. We estimated the effect of each exposure for the entire study period and stratified for the years 2017–2019 and 2020–2021 to account for the COVID-19 pandemic.

Data were analysed using STATA 18.

## Ethical statement

This study did not require external ethical review as its aim was related to analysis of routine surveillance data as part of the core statutory missions of the Finnish Institute for Health and Welfare. As individual patients were not identifiable, no consents were required or obtained.

## Results

During 2017–2021, the Avohilmo database recorded 1 925 176 outpatient visits diagnosed with at least one AURI, of which 982 469 in the public healthcare sector. After excluding prescriptions administered to patients with at least one additional diagnosis justifying an antibacterial prescription, a total of 90 668 antibacterial prescriptions for 90 371 patients were identified. Of those patients, 293 patients had prescriptions for two different antibacterials during a single visit and two patients for three different antibacterials.

In 2017–2019 the annual outpatient visit rate with at least one AURI diagnosis remained stable between 3.8 and 4.7 visits per 100 people (Table 1). From 2019 to 2021 the visit rate decreased from 3.8 to 2.3 visits per 100 people coinciding with the global coronavirus disease 2019 (COVID-19) pandemic. In total, more women (590 265 visits) sought medical care for AURI compared with men (392 200 visits), but the age distribution of the patients was similar between sexes. CPD was the most common underlying diagnosis (12 588 patients), followed by diabetes (971 patients) and chronic inflammatory disease (443 patients). The most frequently diagnosed AURI type was acute upper respiratory infections of multiple and unspecified sites (J06) which comprised 87.3% of all AURI diagnoses.

**Table 1.** Distribution of AURI outpatient visits over time Finland, 2017–2019 (*N* = 1 925 176, % = proportion of public healthcare sector visits)

		Year of visit					
		2017	2018	2019	2020	2021	All
All	*N*	250 037	268 047	224 278	413 253	769 561	1 925 176
Public healthcare sector	*N*	242 877	258 358	212 387	141 481	127 366	982 469
AURI							
J00 Acute nasopharyngitis	*N*	2 767	2 426	1 750	1 405	1 364	9 712
	%	1.1	0.9	0.8	1.0	1.1	1.0
J02 Acute pharyngitis^a^	*N*	12 251	12 835	10 193	7 916	6 619	49 814
	%	5.0	5.0	4.8	5.6	5.2	5.1
J03 Acute tonsillitis^b^	*N*	12 249	11 517	9 024	5 626	4 543	42 959
	%	5.0	4.5	4.2	4.0	3.6	4.4
J04 Acute laryngitis and tracheitis^c^	*N*	7 107	6 878	5 130	2 103	3 116	24 334
	%	2.9	2.7	2.4	1.5	2.4	2.5
J05 Acute obstructive laryngitis (croup) and epiglottitis^d^	*N*	147	138	100	62	87	534
	%	0.1	0.1	<0.1	<0.1	0.1	0.1
J06 AURIs of multiple and unspecified sites^e^	N	208 930	225 248	186 720	124 562	111 862	857 322
	%	86.0	87.2	87.9	88.0	87.8	87.3

*N*, number of visits.

^a^Excluding streptococcal pharyngitis (J02.0) and including ICD-10 codes J02, J02.8, J02.9.

^b^Excluding streptococcal tonsillitis (J03.0) and including ICD-10 codes J03, J03.8, J03.9.

^c^Includes ICD-10 codes J04, J04.0, J04.1, J04.2.

^d^Includes ICD-10 codes J05, J05.0, J05.1.

^e^Includes ICD-10 codes J06, J06.0, J06.8, J06.9.

data source: Avohilmo (Primary Care Outpatient Treatment Register in Finland).

**Figure 2.**
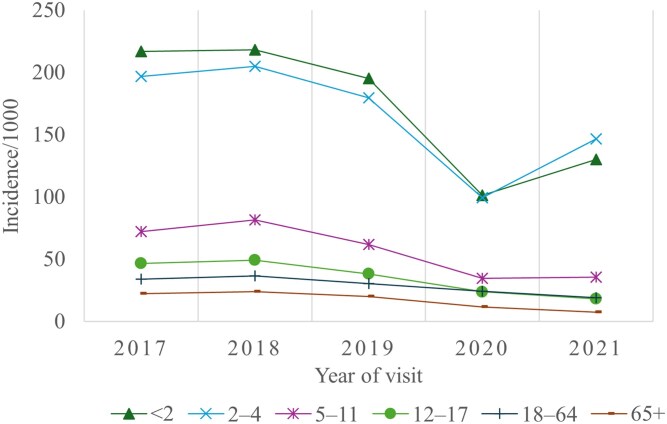
Age^a^ distribution of AURI outpatient visits in the public healthcare sector, Finland, 2017–2021 (*N* = 982 469). (i) ^a^4 missing date of birth. (ii) data source: Avohilmo (Primary Care Outpatient Treatment Register in Finland).

The age distribution displayed a decreasing incidence of visits per 1000 individuals with increasing age (Figure 2). Overall, the highest incidence was among the two youngest age groups. The incidence decreased in all age groups during 2017–2020, with the most notable drop between years 2019 and 2020 in children younger than 2 years and aged 2–4 years, 195–101 per 1000 and 180–99 per 1000, respectively. In 2021 the incidence rose for the two youngest age groups, while the incidence among the three oldest age groups continued decreasing. The highest incidence in the three youngest age groups was among males, whereas in the three oldest age groups the highest incidence was among females.

In the public healthcare sector, roughly 9% of all AURI outpatient visits resulted in an antibacterial prescription (Table 2). There was a decline in the percentage of visits with a prescription from 11.2% in 2017 to 5.5% in 2021. The decreasing trend was observed in all age groups, although it was more noticeable in the three eldest age groups. The percentage of visits resulting in prescriptions rose with increasing age, the highest percentage (15.1%) was detected in those aged 65 years and older. The overall prescription rate was slightly higher for women (9.5%) compared with men (8.8%). As for AURI diagnoses, the highest prescription rate occurred for acute tonsillitis, followed by acute pharyngitis. The prescription rate for AURI visits with an underlying condition ranged from 11%–14% for diabetes, 10%–18% for CPD and 11%–16% for chronic inflammatory disease, with the lower figures detected during the COVID-19 pandemic.

**Table 2.** Percentage of AURI outpatient visits with at least one antibacterial prescription in the public healthcare sector over time and by key factors, Finland, 2017–2021 (*N* = 982 469)

	2017–2019		2020–2021		All	
	Percentage of visits with prescription	95% CI	Percentage of visits with prescription	95% CI	Percentage of visits with prescription	95% CI
All	10.3	0.10–0.10	6.2	0.06–0.06	9.2	0.09–0.09
Age group in years^a^						
<2	2.5	0.02–0.03	2.2	0.02–0.02	2.5	0.02–0.03
2–4	4.6	0.05–0.05	3.1	0.03–0.03	4.2	0.04–0.04
5–11	7.6	0.07–0.08	4.1	0.04–0.04	6.8	0.07–0.07
12–17	12.6	0.12–0.13	8.7	0.08–0.09	11.7	0.11–0.12
18–64	12.6	0.12–0.13	7.0	0.07–0.07	10.9	0.11–0.11
65+	16.3	0.16–0.17	10.8	0.10–0.11	15.1	0.15–0.15
Sex						
Male	9.8	0.10–0.10	5.9	0.06–0.06	8.8	0.09–0.09
Female	10.7	0.11–0.11	6.3	0.06–0.06	9.5	0.09–0.10
AURI						
J00 Acute nasopharyngitis	5.9	0.05–0.07	3.9	0.03–0.05	5.3	0.05–0.06
J02 Acute pharyngitis^b^	31.4	0.31–0.32	21.2	0.20–0.22	28.4	0.28–0.29
J03 Acute tonsilitis^c^	47.6	0.47–0.48	36.3	0.35–0.37	44.9	0.44–0.45
J04 Acute laryngitis and tracheitis^d^	8.4	0.08–0.09	4.0	0.03–0.04	7.5	0.07–0.08
J05 Acute obstructive laryngitis (croup) and epiglottitis^e^	4.5	0.02–0.07	2.0	0–0.04	3.8	0.02–0.05
J06 Acute upper respiratory infections of multiple and unspecified sites^f^	7.3	0.07–0.07	4.1	0.04–0.04	6.4	0.06–0.06

*N*, number of visits.

^a^4 missing date of birth.

^b^Excluding streptococcal pharyngitis (J02.0) and including ICD-10 codes J02, J02.8, J02.9.

^c^Excluding streptococcal tonsillitis (J03.0) and including ICD-10 codes J03, J03.8, J03.9.

^d^Includes ICD-10 codes J04, J04.0, J04.1, J04.2.

^e^Includes ICD-10 codes J05, J05.0, J05.1.

^f^Includes ICD-10 codes J06, J06.0, J06.8, J06.9.

data source: Avohilmo (Primary Care Outpatient Treatment Register in Finland) and Kela (Finnish Social Insurance Institution).

Overall, in the univariate analysis, age under 12 years was associated with the lowest (age <2 years, adjusted probability: 0.21; 95%CI 0.20–0.22; 2–4 years, adjusted probability: 0.36; 95%CI 0.35–0.37; 5–11 years, adjusted probability: 0.59; 95%CI 0.58–0.61), and age >65 years with the highest likelihood of obtaining a prescription (adjusted probability 1.45; 95%CI 1.42–1.48), compared with adults aged 18–64 years. Women were more likely to receive an antibacterial prescription for AURI compared with men (adjusted probability 1.09; 95%CI 1.08–1.11). During the pandemic years 2020–2021, the likelihood of obtaining a prescription in general decreased. However, adolescents aged 12–17 years (adjusted probability 1.27; 95%CI 1.19–1.34) and adults aged >65 years (adjusted probability 1.60; 95%CI 1.53–1.68) obtained prescriptions more often than before the pandemic years.

After adjusting for age, sex, year and AURI type, CPD was the only underlying disease associated with an increased likelihood of prescribing (adjusted probability 1.93; 95%CI 1.84–2.03). The AURI types J03 (adjusted probability 14.70; 95%CI 13.41–16.12) and J02 (adjusted probability 6.97 95%CI 6.35–7.64) were associated with the highest likelihood of antibacterial prescribing in the multivariable model.

## Discussion

Overall, 9% of all patients with AURI diagnoses, were prescribed antibacterials in the public primary healthcare sector. This proportion decreased over time but increased with age. Patients with CPD had a greater likelihood of being prescribed antibacterial medication for an AURI. Patients with J03 (acute tonsillitis) and J02 (acute pharyngitis) were prescribed antibacterials considerably more frequently than those with other AURI types.

The Finnish AMC surveillance data reported to ESAC-Net is based on national sales statistics. However, these data do not allow distinguishing outpatient AMC from hospital consumption in a reliable way.^20^ In 2021 the overall consumption of antibacterials in the community and hospital sector was 11.3 DDD per 1000 inhabitants per day in Finland, which is lower than in Denmark at 14.4 DDD and Norway at 14.0 DDD but higher than in Sweden at 10.1 DDD per 1000 inhabitants per day according to ESAC-Net. The consumption of beta-lactamase sensitive penicillins, for example phenoxymethylpenicillin and benzylpenicillin, is higher in other Nordic countries (Denmark 3.1 DDD, Norway 2.5 DDD, Sweden 2.1 DDD per 1000 inhabitants per day) than in Finland (0.9 DDD per 1000 inhabitants per day). First generation cephalosporins are more widely used in Finland, at 1.5 DDD, compared with all other countries in Europe (EU/EEA crude population-weighted mean 0.15 DDD per 1000 inhabitants per day). This could be attributed to the difference in national outpatient treatment guidelines, for example, in Finland cephalexin is stated as one of the first-line treatment options for skin and soft tissue infections.^21^

In our study, during 2017–2019 the prescription rate for all AURI diagnoses was 10.3%. This is smaller than rates reported in similar studies. A report from China in 2017–2019 reported that 55.1% of patients with AURI were prescribed antibacterials,^22^ however, in contrast to our study, this prescription rate included bacterial diagnoses J02.0 streptococcal tonsillitis and J03.0 streptococcal pharyngitis. On the other hand, a study from the USA from June 2017 to May 2018,^23^ observed prescription rates of 31.9% and 25.9% for non-specific AURI (J06) and pharyngitis (J02), respectively. In our data, the prescription rate for J02 was 31.4% and J06 was 7.3% in 2017–2019.

We also noted a decrease in the overall antibacterial prescription rate for an AURI from 11.2% in 2017 to 9.7% in 2019. This decrease has also been noted in previous studies observing outpatient antibacterial use in Finland.^24,25^ The favourable trend in the antibacterial prescription rate in pre-pandemic years might be attributed to antibacterial campaigns, yearly promotion of the European Antibiotic Awareness Day and national guidelines deterring the use of antibacterials in AURIs.^26^ Improving the surveillance and statistics of antimicrobial surveillance is also included in the national action plan on AMR implemented in 2017.^9^

We observed even more of a decrease in the antibacterial prescription rate for an AURI from 10.3% in the pre-pandemic years 2017–2019 to 6.2% in the pandemic years 2020–2021. This coincided with the reduction in AURI outpatient visits: 258 000 versus 127 000 visits per year, respectively. A similar decrease in antibacterial prescribing for upper respiratory tract infections has been reported previously in other studies observing the effects of COVID-19 in Finland.^27,28^ This decrease occurred most probably because of the reduction of in community circulation of all non-SARS-CoV-2 respiratory viruses during the pandemic,^29,30^ leading to a low incidence of AURIs.

We observed that older people were more likely to receive an antibacterial prescription for an AURI. This observation is in line with reports from the USA but differs from experience from Norway and Japan.^23,31,32^ The higher prescription rate could be attributed to doctors assuming an increased risk of secondary bacterial infections among older age groups, and due to an earlier national recommendation advising antibacterial therapy for bronchitis in older patients.^33^ The prescription rate for an AURI also increased with age among children: children <2 years of age were least likely to obtain an antibacterial prescription for an AURI. A similar finding was reported by Finnish colleagues who reported that younger age was associated with a smaller likelihood for antibacterial prescribing in children with an AURI in the private healthcare sector in Finland in 2014–2020.^13^

In all age groups, patients with acute tonsillitis or acute pharyngitis, were more likely to receive an antibacterial prescription, even after adjusting for demographic factors, year and underlying conditions. This occurred despite excluding the specific ICD-10 codes indicating a bacterial aetiology of tonsillitis and pharyngitis. One explanation might be that the initial visit is coded as unspecified acute tonsillitis (J03.9), which is not corrected afterwards even if streptococcal infection would be confirmed and antibacterial treatment initiated.

Our register-based study had several limitations. First, the prescription data did not allow analysis of dosage or length of therapy, thus it was not possible to estimate the total antibacterial consumption related to AURIs. Second, underlying conditions were considered only if the corresponding diagnosis code was recorded for the AURI visit. Furthermore, we explored only a few underlying conditions to explain antibacterial consumption, and the ICD-10 code does not confer disease severity or timing, and hence, the true effect of the underlying disease was not likely to be similar for all cases with the same diagnosis. Moreover, our disease data did not contain primary diagnosis information if a patient had more than one diagnosis recorded during a visit. Nevertheless, we eliminated any associated prescriptions when an additional diagnosis that warranted an antibacterial prescription was recorded.

Our study includes data on all Finnish public healthcare sector AURI outpatient visits and prescriptions for all age groups and provides new evidence on the appropriateness of antibacterial prescribing in Finland. Our research indicates that AURI diagnoses, most of which are of viral origin, are still leading to antibacterial prescribing in all age groups. The prescribing habits appear to be influenced by patient demographics and underlying conditions. However, the prescription rate seems to be decreasing. Further investigations to understand behavioural patterns behind antibacterial prescribing are necessary for enhancing antimicrobial stewardship initiatives and controlling inappropriate antimicrobial prescriptions, thereby mitigating the risk of adding to AMR.
